# Congenital cytomegalovirus infection and the risk of hearing loss in childhood: A PRISMA-compliant meta-analysis

**DOI:** 10.1097/MD.0000000000027057

**Published:** 2021-09-10

**Authors:** Pei-Hui Liu, Jin-Dou Hao, Wei-Yan Li, Jia Tian, Jie Zhao, Yong-Mei Zeng, Guo-Qing Dong

**Affiliations:** Department of Pediatrics, Affiliated Shenzhen Maternal & Child Healthcare Hospital of Southern Medical University, Shenzhen, China.

**Keywords:** congenital cytomegalovirus infection, hearing loss, meta-analysis

## Abstract

**Background:**

Congenital cytomegalovirus (cCMV) infection is the most common cause of childhood hearing loss (HL), although the strength of this association remains limited and inconclusive. Thus, the purpose of this study was to summarize evidence regarding the strength of the relationship between cCMV and childhood HL and to determine whether this relationship differs according to patient characteristics.

**Methods:**

The PubMed, EmBase, and Cochrane Library databases were searched for studies evaluating the relationship between cCMV and HL from inception to September 2019. Odds ratios (ORs) and corresponding 95% confidence intervals (CIs) were used to calculate the investigated outcomes in a random-effects model. Sensitivity, subgroup, and publication bias analyses were also performed.

**Results:**

A total of 15 studies involving 235,026 children met the inclusion criteria and were included in the final analysis. The summary results indicated that cCMV infection was associated with an increased risk of HL (odds ratio [OR]: 8.45; 95% confidence interval [CI]: 3.95–18.10; *P* < .001), irrespective of whether studies reported sensorineural HL (OR: 5.42; 95% CI: 1.98–14.88; *P* = .001), or did not evaluate HL types among their patients (OR: 11.04; 95% CI: 3.91–31.16; *P* < .001). However, in studies conducted in the United States (*P* < 0.001) and published in or after 2000 (*P* = 0.026), the study populations included <60% males (*P* < 0.001). Moreover, studies of high quality (*P* < .001) demonstrated a significantly greater risk of HL with cCMV infection than that in the corresponding subgroups.

**Conclusions:**

The study results suggest that cCMV infection increases the risk of HL. Further studies are required to investigate the association of cCMV infection with the risk of specific subtypes of HL.

## 1 Introduction

Hearing impairment is a commonly encountered disease affecting the progression of speech and language, which occurs in 1 to 3 infants per 1000 live births in the United States, affecting approximately 4000 to 12,000 infants.^[1]^ A number of factors, including viral infections, microcirculatory disorders, autoimmune disorders, and labyrinthine hemorrhages, have been implicated in the pathophysiology of hearing loss (HL).^[2–5]^ Particularly, HL in adults is primarily caused by microcirculatory disorders, whereas viral infection has been found to be the main cause of HL in children.^[6–8]^

Congenital cytomegalovirus (cCMV) infection is the most frequently occurring congenital viral infection in children, affecting approximately 0.64% to 0.7% of neonates worldwide.^[9]^ Notably, around 90% of cCMV-positive infants have been reported to be asymptomatic at birth; however, 6% to 23% infants later develop sensorineural HL (SNHL).^[10,11]^ Furthermore, the majority of children with cCMV infection remain undiagnosed due to the lack of symptoms at birth and the lack of universal screening for CMV infection at birth and during pregnancy. Given these circumstances, the strength of the relationship between cCMV and SNHL may be underestimated, resulting in the development of late sequelae associated with cCMV.^[12–15]^ Therefore, cCMV and hearing screenings should be assessed concurrently in neonates to identify early-onset HL, and infants who pass initial hearing screening should be regularly monitored to detect late-onset HL.

Two systematic reviews have summarized the importance of cCMV as a cause of childhood SNHL, although the strength of the relationship between cCMV and childhood HL was not reported in both studies.^[16,17]^ Clarifying the association between cCMV and HL is especially important in children. Therefore, in this study, we comprehensively examined the available studies to explore the association between cCMV infection and the risk of HL in children, and compared this relationship between patients with different characteristics.

## 2 Methods

### 2.1 Data sources, search strategy, and selection criteria

This study followed the Preferred Reporting Items for Systematic Reviews and Meta-Analysis Statement issued in 2009 (Checklist S1).^[18]^

Any observational study that investigated the association between cCMV and the risk of HL was eligible for inclusion in the present study, without restrictions on publication language or status. In brief, a literature search was conducted across the PubMed, Embase, and Cochrane library databases with keywords, including ‘cytomegalovirus, “hearing loss," and “congenital," from their inception up to September 2019. A manual search of each study's reference list was performed to identify additional potentially eligible studies. Furthermore, the study topic, design, status of children, exposure, and incidence of HL in each potential study was assessed to identify studies suitable for inclusion.

Following a standardized approach, two independent reviewers conducted the literature search and study selection, and conflicts between them were resolved by discussions until a consensus was reached. The inclusion criteria for studies in this meta-analysis were as follows: participants, children with or without cCMV infection; exposure, cCMV infection; control, no cCMV infection; outcome, incidence of HL; and study design, observational studies regardless of design. In contrast, the exclusion criteria consisted of reviews, editorials, non-human studies, letters, and conference articles without sufficient data.

### 2.2 Data collection and quality assessment

The following items were extracted from the included articles: first author's name, publication year, country, study design, sample size, mean age of participants, proportion of male participants, birth weight of participants, and incidence of HL. The methodological quality of each study was then assessed using the Newcastle-Ottawa Scale (NOS), which is based on three subscales, namely selection (4 items), comparability (1 item), and outcome (3 items), and uses a “star system” (range: 0–9) for quality assessment.^[19]^ As mentioned previously, data extraction and quality assessment were conducted independently by 2 authors, and any disagreement was settled with a review of the original article by the corresponding author of this meta-analysis.

### 2.3 Statistical analysis

The association between cCMV infection and HL risk was examined based on the abstracted incidence of HL and number of cCMV patients in each study, and the pooled odds ratio (OR) and its corresponding 95% confidence interval (CI) were used to calculate the pooled results using a random-effects model.^[20,21]^ A heterogeneity test for the investigated outcome was conducted across the included studies using *I*^*2*^ and *Q* statistics, and *P* < .10 was considered significantly heterogeneous.^[22,23]^ Sensitivity analysis was used to explore the source of heterogeneity and to evaluate the influence of a single study on the pooled conclusion.^[24]^ In addition, subgroup analyses were conducted according to publication year, country, study design, sample size, proportion of male participants, HL types, and study quality. Based on the ORs and 95% CIs in each subset, the ratios of ORs with 95% CIs were calculated between subgroups, and *P* values for the heterogeneity between subgroups were calculated using the *χ*^2^ test.^[25]^ Furthermore, publication bias was assessed using funnel plots, Egger test, and Begg test.^[26,27]^ Two-tailed *P* values were obtained, and *P* < .05 was considered statistically significant. The STATA software (version 10.0; Stata Corporation, College Station, TX) was used to conduct all analyses in this study.

### 2.4 Ethics and informed consent of patients

Ethics committee approval and informed consent of patients were not required, as this study did not involve confidential patient information.

## 3 Results

### 3.1 Search of the published literature

**Figure 1:**
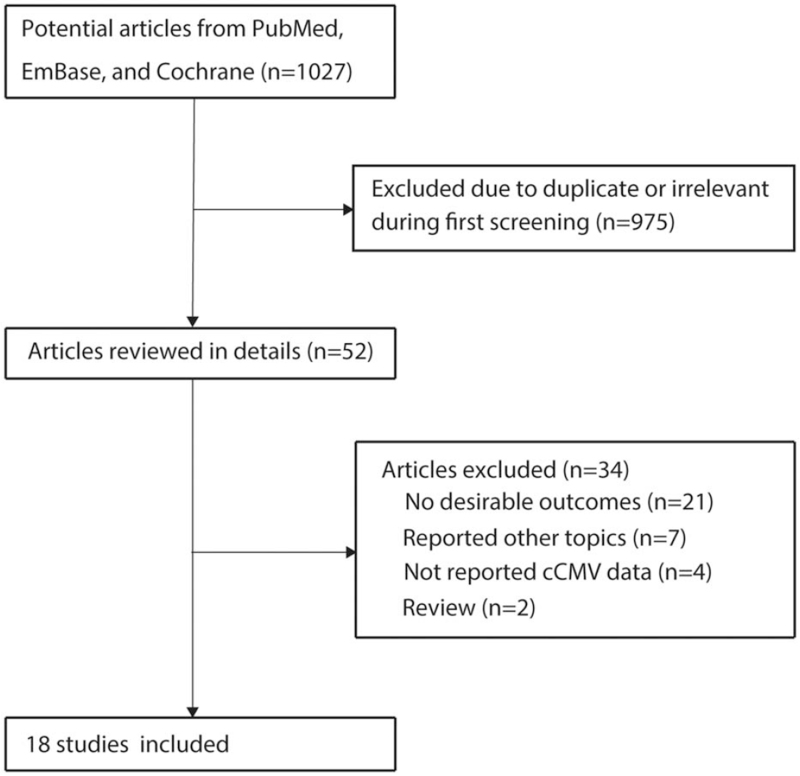
Study selection process.

Initial electronic searches yielded 1027 articles, of which 975 were excluded as duplicate, irrelevant, and containing other design topics. Full texts of the remaining 52 studies were retrieved for evaluation, and a total of 18 studies met the inclusion criteria.^[28–45]^ No additional study was identified through a review of the reference lists of these studies (Fig. 1). Table 1 summarizes the baseline characteristics of identified studies and recruited patients.

**Table 1:** Baseline characteristic of studies included in the systematic review and meta-analysis.

First author	Publication year	Country	Study design	Sample size	Mean age, wk	Proportion of male participants (%)	Birth weight, g	Reported outcomes	NOS score
Harris et al^[28]^	1984	Sweden	Prospective	94	NA	NA	NA	SNHL	7
Saigal et al^[29]^	1982	Canada	Prospective	93	39.0	63.4	3125	SNHL	7
Kumar et al^[30]^	1984	USA	Prospective	38	NA	NA	NA	SNHL	6
Dar et al^[31]^	2017	India	Prospective	1720	37.6	45.8	2700	HL	8
Boppana et al^[32]^	1992	USA	Prospective	106	NA	51.0	NA	HL	6
Fowler et al^[33]^	1997	USA	Retrospective	508	NA	48.8	NA	SNH	7
Samileh et al^[34]^	2008	Iran	Retrospective	152	150.0	63.8	NA	SNHL	6
Peckham et al^[35]^	1987	UK	Prospective	120	NA	NA	NA	SNHL	7
Diener et al^[36]^	2017	US	Prospective	1078	NA	NA	NA	HL	6
Bradford et al^[37]^	2005	USA	Prospective	40	2.0	67.5	NA	HL	6
Fowler et al^[38]^	2017	USA	Prospective	99,945	NA	50.8	NA	HL	7
Hicks et al^[39]^	1993	USA	Retrospective	2036	NA	NA	NA	HL	7
Rosenthal et al^[40]^	2009	USA	Prospective	1731	NA	NA	NA	HL	7
Yamamoto et al^[41]^	2011	USA	Prospective	85	NA	57.6	NA	HL	8
Turner et al^[42]^	2014	USA	Retrospective	198	NA	NA	NA	SNHL	7
Lanzieri et al^[43]^	2017	USA	Retrospective	143	NA	60.8	NA	SNHL	6
Dumanch et al^[44]^	2017	USA	Retrospective	11,5039	NA	NA	NA	HL	7
Yamamoto et al^[45]^	2019	Brazil	Prospective	11,900	NA	51.9	3180	HL	7

### 3.2 Study characteristics

Of the 18 included studies, 12 were prospective in design,^[28–32,35–38,40,41,45]^ and 6 were retrospective studies.^[33,34,39,42–44]^ The sample size ranged from 38 to 115,039 participants, and the proportion of male participants ranged from 45.8% to 67.5%. Regarding the country of publication, 12 studies were conducted in the United States,^[30,32,33,36–44]^ and 1 study each was reported from Sweden, Canada, India, Iran, the United Kingdom, and Brazil.^[28,29,31,34,35,45]^ Regarding HL data, SNHL data were available in 8 studies,^[28–30,33–35,42,43]^ and HL data were available in 10 studies.^[31,32,36–41,44,45]^ Regarding study quality, which was evaluated using NOS, studies receiving ≥7 stars were regarded as high-quality studies. Two studies obtained a score of 8,^[31,41]^ 10 studies obtained a score of 7,^[28,29,33,35,38–40,42,44,45]^ and 6 studies obtained a score of 6.^[30,32,34,36,37,43]^

### 3.3 Meta-analysis

**Figure 2:**
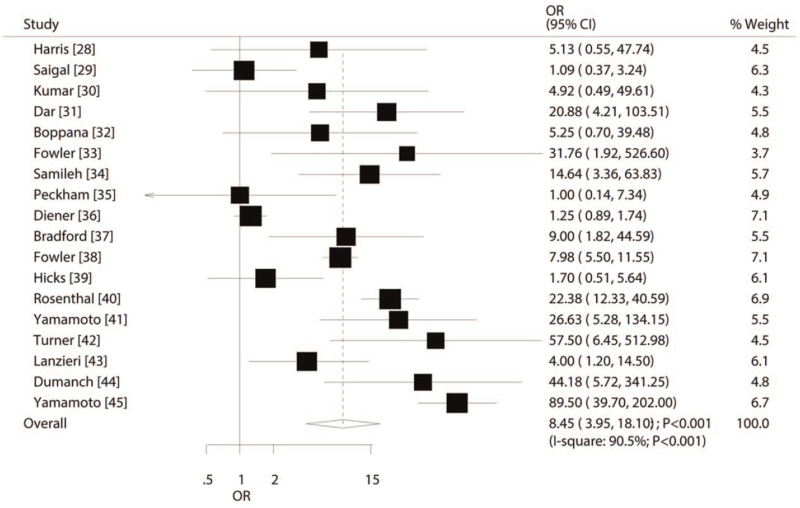
Association between congenital cytomegalovirus infection and the risk of hearing loss.

**Figure 3:**
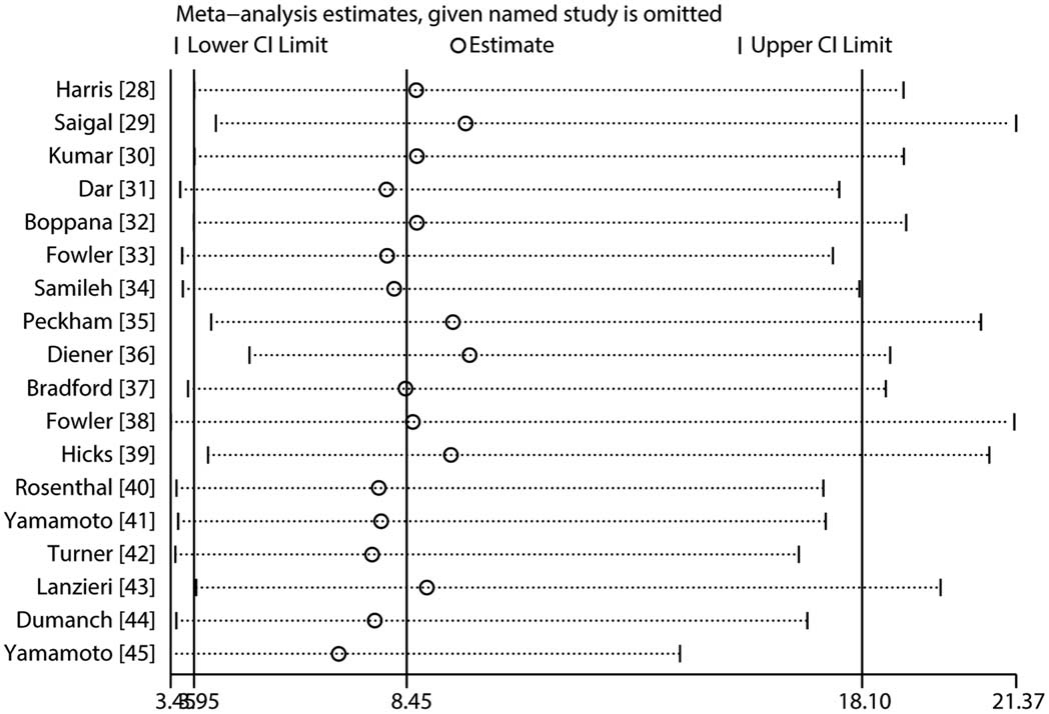
Sensitivity analysis for the relationship between congenital cytomegalovirus infection and the risk of hearing loss.

All included studies reported an association between cCMV infection and HL risk. Specifically, the pooled OR indicated that cCMV-positive participants had a significantly higher risk of developing HL as compared to cCMV-negative participants (odds ratio [OR], 8.45; 95% confidence interval [CI], 3.95–18.10; *P* < .001; Fig. 2). Moreover, there was significant heterogeneity across the included studies (*I*^*2*^ = 90.5%; *P* < .001), and the sensitivity analysis showed that the conclusion was not altered by excluding any individual study (Fig. 3).

### 3.4 Subgroup analyses

Subgroup analyses of HL risk, including publication year, country, study design, sample size, proportion of male participants, reported outcomes, and study quality, were performed to explore the source of heterogeneity and evaluate the relationship between cCMV infection and HL risk in patients with specific characteristics (Table 2). Notably, the heterogeneity between subgroups based on publication year (*P* = .004), country (*P* < .001), proportion of male participants (*P* < .001), and study quality (*P* < .001) was statistically significant. Moreover, cCMV-positive participants were found to have a significantly increased risk of developing HL as compared to cCMV-negative participants in all subgroups. Furthermore, studies published in or after 2000 reported a significantly greater risk of HL than those published before 2000 (ratio of OR, 6.18; 95% CI, 1.80–21.23). Furthermore, studies conducted in the United States; retrospective studies; high-quality studies; and studies with a sample size ≥100, a proportion of male participants <60%, or HL as the outcome reported a greater risk of HL than that in corresponding subgroups, although these differences were not statistically significant.

**Table 2:** Subgroup analyses.

Subgroup	OR and 95% CI	*P*	Heterogeneity(%)	Between-subgroup heterogeneity	Ratio between subgroups
Publication year					
2000 or After	14.58 (5.44–39.14)	<.001	93.8	0.004	6.18 (1.80–21.23)
2000 Previous	2.36 (1.12–4.94)	.023	20.9		
Country					
USA	8.50 (3.60–20.08)	<.001	90.2	<0.001	1.10 (0.15–7.85)
Other	7.71 (1.32–45.00)	.023	89.8		
Study design					
Prospective	7.28 (2.82–18.84)	<.001	93.2	0.258	0.66 (0.14–3.01)
Retrospective	11.08 (3.38–36.35)	<.001	66.3		
Sample size					
≥100	9.83 (3.93–24.58)	<.001	92.8	0.524	1.80 (0.37–8.81)
<100	5.46 (1.49–19.95)	.010	66.3		
Proportion of male participants (%)					
≥60.0	4.50 (1.38–14.65)	.013	68.2	<0.001	0.22 (0.04–1.16)
<60.0	20.40 (6.32–65.83)	<.001	83.6		
Reported outcomes					
SNHL	5.42 (1.98–14.88)	.001	61.7	0.441	0.49 (0.12–2.09)
HL	11.04 (3.91–31.16)	<.001	94.4		
Study quality					
High	11.26 (4.92–25.74)	<.001	84.9	<0.001	2.51 (0.67–9.42)
Low	4.49 (1.60–12.63)	.004	74.3		

### 3.5 Publication bias

**Figure 4:**
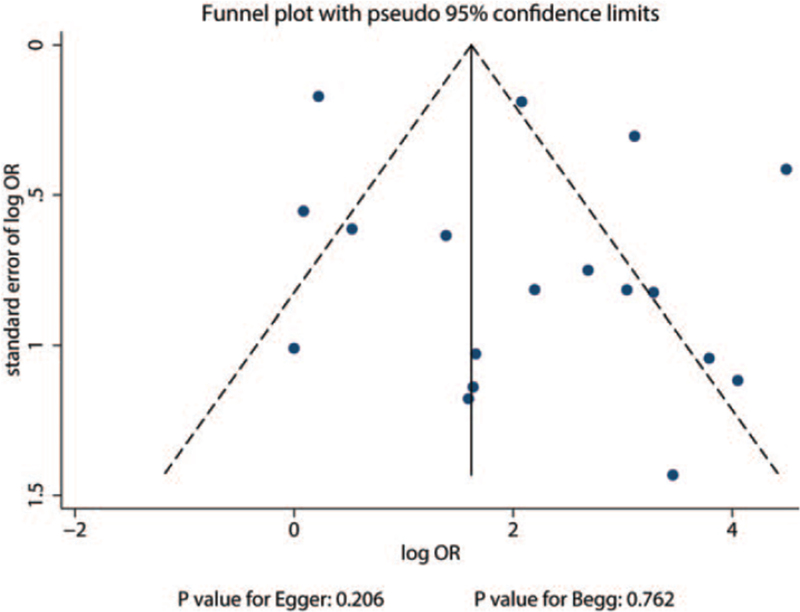
Evaluation of publication bias.

Publication bias could not be ruled out by reviewing the funnel plot (Fig. 4). The results of Egger and Begg tests revealed no significant publication bias (Egger, *P* = .206; Begg, *P* = .762).

## 4 Discussion

Since the characteristics of cCMV infection may affect the incidence of HL in children, the correlation between cCMV infection and HL risk was evaluated according to publication year, country, study design, sample size, proportion of male participants, reported outcomes, and study quality. This meta-analysis included a total of 235,026 participants from 12 prospective and 6 retrospective studies, and the results indicated that cCMV infection was associated with an increased risk of HL. Furthermore, the conclusions of subgroup analyses were consistent with those of the overall analysis for the most part; however, publication year, country, proportion of male participants, and study quality could have possibly biased the association between cCMV and HL risk.

A previous systematic review of 37 studies concluded that the prevalence of cCMV in developed countries was 0.58% (95% CI, 0.41–0.79). The report also indicated that 12.6% (95% CI, 10.2–16.5) of cCMV-positive children developed HL, of which 33.3% were symptomatic, and 10% were asymptomatic.^[16]^ Moreover, Korver et al (2011) suggested that the causes of childhood hearing impairment were either hereditary (38.9%), acquired (29.7%), miscellaneous (7.1%), or unknown (24.3%) in origin;^[17]^ however, the relationship between cCMV infection and HL risk in children with different characteristics was not illustrated. The major strength of the present study was the comprehensive inclusion of observational studies with a large sample size and broad participant characteristics. The large sample size ensured the reliability of our conclusions, and the broad participant characteristics ensured the applicability of our results.

The pooled results indicated that cCMV-positive individuals had a significantly increased risk of developing HL, although the results were inconsistent between included studies. Harris et al (1984) suggested that cCMV screening in newborns could identify high-risk infants and facilitate the implementation of early intervention strategies. However, they found that the relationship between cCMV infection and SNHL risk was not statistically significant.^[28]^ Similarly, Saigal et al (1982) found that the prevalence of SNHL was similar in cCMV-positive and cCMV-negative groups.^[29]^ Kumar et al (1984) found that asymptomatic CMV infection could result in audiologic sequelae, although this relationship was not statistically significant,^[30]^ whereas Boppana et al (1992) reported that cCMV infection was associated with multi-system disease in newborns, but this did not affect the incidence of HL.^[32]^ Peckham et al (1987) reported that the prevalence of CMV infection was similar in children with and without SNHL.^[35]^ Diener et al (2017) indicated that CMV testing could be used as an adjunct to the newborn hearing screening program to attain timely audiological diagnostics within 90 days, although the risk of HL was not statistically significant.^[36]^ Lastly, Hicks et al (1993) indicated that cCMV infection may be a principal cause of hearing impairment; however, this association was not statistically significant.^[39]^ This study had a smaller sample size and a lower incidence of HL, which may have contributed to its low statistical power and broader 95% CI. Furthermore, the prevalence of asymptomatic cCMV infection across studies was variable, which could have affected subsequent HL risk, although the pooled analysis was not stratified according to asymptomatic or symptomatic cCMV infection. The potential pathogenesis of HL in children with cCMV infection could be attributed to injuries to the endolymphatic structures and stria vascularis, consequently inducing the degeneration of sensory structures.^[46,47]^ Additionally, the occurrence of HL could also be attributed to the cytopathic effect of CMV and host immune responses in the inner ear.^[48]^

For the most part of this study, the results of the stratified analysis were consistent with those of the overall analysis. However, five breakthroughs should be highlighted. First, studies published in or after 2000 reported a higher risk of HL than that in studies published before 2000. This discrepancy may be due to the use of different screening methods, which may have affected the accuracy of the reported incidence of cCMV infection and HL risk. Second, the incidence of HL in relation to cCMV infection was affected by country, which was possibly due to the variations in cCMV prevalence and presentation. Third, a sex difference in the prevalence of HL may exist due to infection with other viruses. Fourth, the study quality may have affected the reported risk of HL due to the accuracy of intrinsic relationships. Lastly, the different number of studies included in each subset may have been correlated with statistical power, and thus summary results may have varied.

This study had certain limitations. First, the ascertainment of cCMV infection rate in each study was inconsistent, which may have introduced confounders to the representative study cohort. Second, recall and selection biases are inevitable in retrospective studies. Third, the majority of the results were calculated with crude data, and numerous factors were not adjusted for correlation with HL risk. Fourth, the substantial heterogeneity observed could not be fully interpreted due to the lack of several important factors. Finally, publication bias and the availability of individual data sets are inherent limitations of all study-level meta-analyses.

In conclusion, cCMV infection increased the risk of HL. Furthermore, in this meta-analysis, study publication year, gender, and study quality affected the apparent risk of developing HL. Further studies should be conducted to clarify the associations between cCMV infection and different subtypes of HL, and to assess the symptoms, diagnosis, and treatments of HL related to cCMV.

## Author contributions

PHL and GQD were involved in the literature search, study design, data collection, data analysis, data interpretation, and preparation of the manuscript. JDH, WYL, JT, JZ, and YMZ conducted the data collection and analysis, and critically revised the manuscript. All authors read and approved the final manuscript.

**Conceptualization:** Pei-Hui Liu, Guo-Qing Dong.

**Data curation:** Pei-Hui Liu, Jin-Dou Hao.

**Formal analysis:** Pei-Hui Liu.

**Investigation:** Jin-Dou Hao, Wei-Yan Li, Jia Tian, Jie Zhao, Yong-Mei Zeng.

**Methodology:** Wei-Yan Li, Jia Tian, Jie Zhao, Yong-Mei Zeng.

**Software:** Guo-Qing Dong.

**Supervision:** Guo-Qing Dong.

**Writing – original draft:** Pei-Hui Liu.

**Writing – review & editing:** Guo-Qing Dong.
